# Two target genes based multiple cross displacement amplification combined with a lateral flow biosensor for the detection of *Mycobacterium tuberculosis* complex

**DOI:** 10.1186/s12866-021-02328-6

**Published:** 2021-10-04

**Authors:** Junfei Huang, Ziyu Xiao, Xinggui Yang, Xu Chen, Xiaojuan Wang, Yijiang Chen, Wenlin Zheng, Wei Chen, Huijuan Chen, Shijun Li

**Affiliations:** 1 Laboratory of Infectious Disease of Experimental Center, Guizhou Provincial Center for Disease Control and Prevention, 73 Bageyan Road, Guiyang, 550004 Guizhou People’s Republic of China; 2 Public Health School, Guizhou Medical University, Guiyang, 550025 Guizhou People’s Republic of China; 3 The Second Affiliated Hospital, Guizhou University of Traditional Chinese Medicine, Guiyang, Guizhou 550003 People’s Republic of China

**Keywords:** *Mycobacterium tuberculosis* complex, Multiple cross displacement amplification, Lateral flow biosensor, Detection, Diagnosis

## Abstract

**Background:**

Tuberculosis (TB) is a serious chronic infectious disease caused by *Mycobacterium tuberculosis* complex (MTBC). Hence, the development of a novel, simple, rapid and sensitive method to detect MTBC is of great significance for the prevention and treatment of TB.

**Results:**

In this study, multiple cross displacement amplification (MCDA) combined with a nanoparticle-based lateral flow biosensor (LFB) was developed to simultaneously detect two target genes (*IS6110* and *mpb64*) of MTBC (MCDA-LFB). One suite of specific MCDA primers designed for the *IS6110* and *mpb64* genes was validated using genomic DNA extracted from the reference strain H37Rv. The MCDA amplicons were analyzed using a real-time turbidimeter, colorimetric indicator (malachite green, MG) and LFBs. The optimal amplification temperature and time were confirmed, and the MCDA-LFB method established in the current report was evaluated by detecting various pathogens (i.e., reference strains, isolates and clinical sputum samples). The results showed that the two sets of MCDA primers targeting the *IS6110* and *mpb64* genes could effectively detect MTBC strains. The optimal reaction conditions for the MCDA assay were determined to be 67 °C for 35 min. The MCDA assay limit of detection (LoD) was 100 fg per reaction for pure genomic DNA. The specificity of the MCDA-LFB assay was 100%, and there were no cross-reactions for non-MTBC strains. For sputum samples and MTBC strain detection, the positive rate of MCDA-LFB for the detection of MTBC strains was consistent with seminested automatic real-time PCR (Xpert MTB/RIF) and higher than acid-fast staining (AFS) and culture assays when used for sputum samples. The MCDA-LFB assay was a rapid tool, and the whole procedure for MCDA-LFB, including DNA template preparation, MCDA reaction and amplification product analysis, was completed within 70 min.

**Conclusion:**

The MCDA-LFB assay targeting the *IS6110* and *mpb64* genes is a simple, rapid, sensitive and reliable detection method, and it has potential significance for the prevention and treatment of TB.

## Background

Tuberculosis (TB) is a chronic infectious disease caused by *Mycobacterium tuberculosis* complex (MTBC), and the MTBC pathogens evolve *M. tuberculosis, M. bovis, M. bovis Bacillus Calmette-Guerin* (BCG), *M. africanum*, *M. carinii*, *M. suricattae*, *M. orygis*, *M. microti*, *M. caprae*, *M. mungi*, *M. canettii, M. pinnipedii*, and *M*. *vole*. In particular, *M. tuberculosis*, *M. bovis* and *M. africanum* are highly pathogenic bacteria [1–4]. TB seriously endangers human health and is a public health and social problem of global concern [5]. The World Health Organization (WHO) has listed TB as one of the major infectious diseases. In 2018, there were approximately 10 million new TB cases and 1.5 million TB-related deaths worldwide [5]. Approximately one-third of the world’s people have been and/or are now infected with MTB, according to estimates from the World Health Organization [6, 7]. Thus, quick and accurate strategies for the detection and identification of MTBC strains are important for the prevention and treatment of TB. In general, the diagnosis methods for TB usually rely on conventional sputum smear microscopy (SSM) and culture identification of the organism (namely, mycobacterial cultivation identification and biochemical tests). However, deficiencies for the abovementioned conventional examination methods (including low sensitivity, time-consuming, and complicated operation steps) cannot meet the requirements for rapid and specific detection of MTBC. Hence, simple, fast, accurate and reliable detection methods are required for the detection of MTBC in laboratory diagnosis [6, 8]. With the development of molecular techniques, polymerase chain reaction (PCR) and PCR-based assays (e.g., real-time PCR, nested PCR, and GeneXpert) have been widely used for the diagnosis of TB [9–12]. Although PCR and PCR-based assays have excellent sensitivity and reliability, the needs for specific instruments and/or reagents hinder their application in basic laboratories. To overcome the shortcomings of PCR techniques, isothermal amplification techniques, including loop-mediated isothermal amplification (LAMP), cross-priming isothermal amplification (CPA), and multiple cross displacement amplification (MCDA), were developed and applied for the diagnosis of TB [13–15]. In particular, the MCDA assay based on the isothermal strand-displacement polymerization reaction, which is a highly specific and sensitive detection technique, has been established and implemented in previous studies [16, 17]. The target sequence has ten special primers spanning ten distinct regions and requires a constant temperature to react [16–18]. Real-time turbidity, agarose gel electrophoresis, colorimetric indicators and nanoparticle-based lateral flow biosensors (LFBs) were selected for MCDA product analysis, especially MCDA combined with nanoparticle-based LFB (MCDA-LFB), which makes product analysis simple and visual [15–19]. The MCDA technique was applied for the detection of *Listeria monocytogenes* [18], *Salmonella* spp. strains and *Shigella* spp. [19] and *Vibrio parahaemolyticus* [20]. Our team also created the MCDA method for *Brucella* spp. [17] and *Neisseria meningitidis* [16] detection successfully.

In this study, the MCDA-LFB method for MTBC detection was successfully established by our group. Two specific target genes, *IS6110* and *mpb64*, were chosen for MTBC-MCDA detection, and 10 specific primers were designed for the targets. The MCDA products were analyzed using LFB, real-time turbidity and colorimetric indicators (malachite green, MG). Then, the MCDA reaction conditions, including amplification temperature and time, were optimized. Subsequently, the sensitivity and specificity of the MTBC-MCDA-LFB technique were tested, and then MCDA-LFB was applied to detect sputum specimens and MTBC strains from clinical samples.

## Results

### Confirmation and detection of MCDA products

**Fig. 1:**
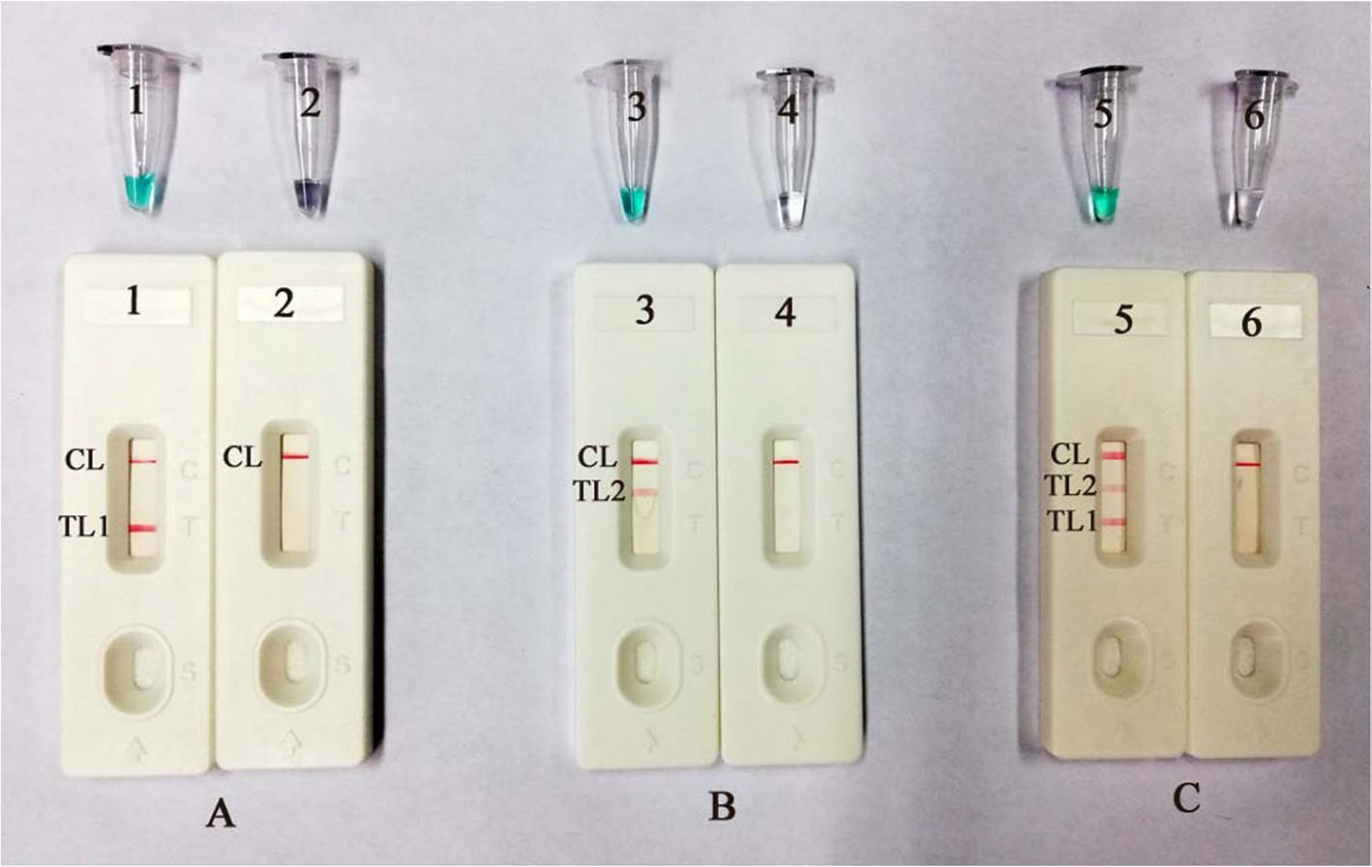
Confirmation and detection of MTBC-MCDA products. **A** The LFB and MG methods were applied for *IS6110* gene amplification. The products of the MTBC-MCDA assay were visually analyzed by observation of TL1 and color change. Tube 1/biosensor 1: positive amplification of *M. tuberculosis*; Tube 2/biosensor 2: black control of DW. **B** The LFB and MG methods were applied for *mpb64* gene amplification. The products of the MTBC-MCDA assay were visually analyzed by observation of TL2 and color change. Tube 3/biosensor 3: positive amplification of *M. tuberculosis*; Tube 4/biosensor 4: black control of DW. **C** The LFB and MG methods were applied for both *IS6110* and *mpb64* gene amplification. The products of the MTBC-MCDA assay were visually analyzed by observation of TL1, TL2 and color change. Tube 5/biosensor 5: positive amplification of *M. tuberculosis*; Tube 6/biosensor 6: black control of DW

The disposable lateral flow biosensor (LFB) consists of test Line 1 (TL1), test Line 2 (TL2) and a control line (CL). First, the *IS6110* gene (Fig. 1A) and *mpb64* gene (Fig. 1B) were examined by MCDA amplification and detected with LFB and MG, respectively. Then, both target genes were detected at the same time (Fig. 1C). Genomic templates of the standard strain *M. tuberculosis* (H37Rv, ATCC 27294) were used for the MCDA assay.

### Optimization of the temperatures for the MTBC-MCDA assay

**Fig. 2:**
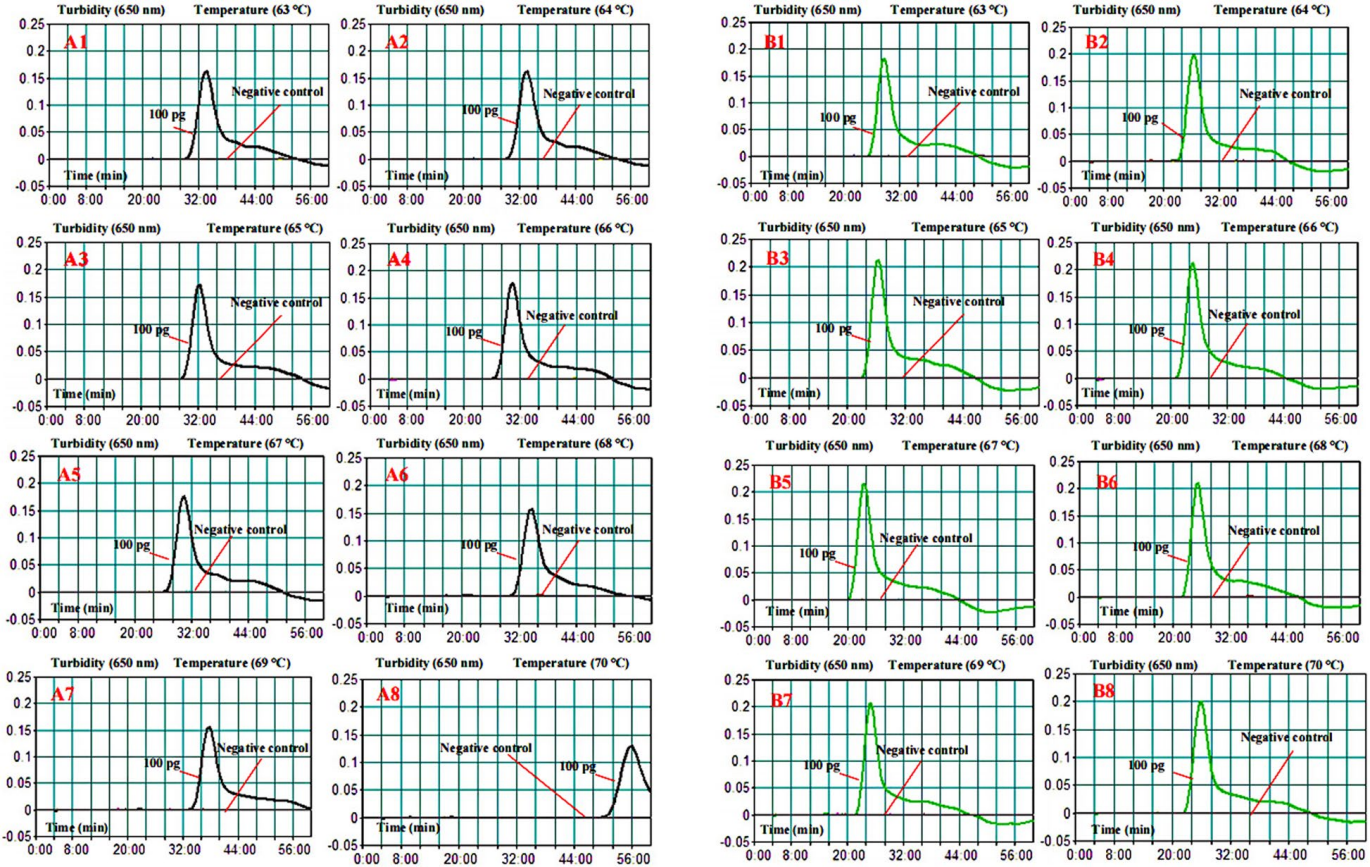
Optimization of reaction temperature for MTBC-MCDA primers. Both the *IS6110* gene (**A1-A8**) and *mpb64* gene (**B1-B8**) were detected at different temperatures. The genes were detected for different temperatures (63–70 °C, 1 °C intervals) with template DNA at the level of 100 pg per reaction. The standard MCDA reactions for the detection of *M. tuberculosis* were monitored by a real-time turbidimeter, the threshold value was 0.1, and a turbidity > 0.1 was set as positive. The *M. avium* genomic templates were used as a negative control

To evaluate the optimum amplification temperature, *M. tuberculosis* (H37Rv, ATCC 27294) strain genomic templates were used as the positive controls at a level of 100 pg per reaction, and the reactions were monitored by the real-time turbidity (LA-320C) method. Both the *IS6110* gene and *mpb64* gene were detected, and the effect was examined at fixed temperatures ranging from 63 to 70 °C with 1 °C intervals for MCDA amplification. The *M. avium* genomic templates were used as negative controls. According to Table 1 and Fig. 2, at 67 °C, both *IS6110* and *mpb64* gene amplification had the shortest time with higher turbidity and faster reaction times at 30 and 23 min. Thus, an amplification temperature of 67 °C was applied to perform the remaining experiments in the study.

**Table 1:** Reaction temperature optimization for MTBC-MCDA primers

Temperature (°C)	***IS6110*** gene		***mpb64*** gene	
	Peak of turbidity	Time (min)	Peak of turbidity	Time (min)
63	0.16	34	0.18	29
64	0.14	31	0.20	27
65	0.17	32	0.21	26
66	0.18	30	0.21	25
67	0.18	30	0.22	23
68	0.16	34	0.21	25
69	0.15	38	0.20	25
70	0.13	55	0.20	26

The threshold of turbidity> 0.1 was judged as positive for the MCDA reaction by a real-time turbidimeter

### Optimization of reaction time for the MTBC-MCDA-LFB assay

**Fig. 3:**
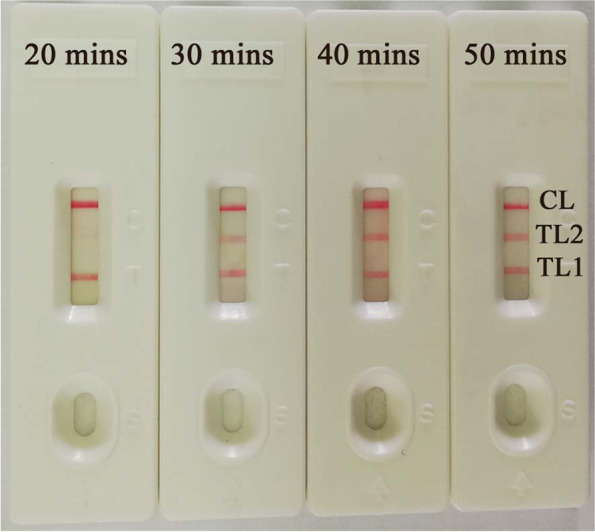
The optimal duration of time required for the MTBC-MCDA-LFB method. Four distinct reaction times (20, 30, 40, and 50 min) were examined and compared at 67 °C. Biosensors represent DNA levels of 100 pg of *M. tuberculosis* templates

The four reaction times (20, 30, 40 and 50 min) were tested at 67 °C according to the standard MCDA conditions for the optimum time by the MTBC-MCDA-LFB assay during the reaction stage. The DNA level with 100 pg of *M. tuberculosis* genomic templates per reaction was displayed by three visible-red lines (TL1, TL2 and CL) on the LFB. The earliest test lines were observed for both TL1 and TL2 when the amplification lasted for 30 min (the threshold of turbidity> 0.1) (Fig. 3). To ensure adequate amplification, a reaction time of 35 min was recommended as a reasonable reaction time for the MTBC-MCDA-LFB assay in this research.

### Sensitivity of the MTBC-MCDA-LFB assay

**Fig. 4:**
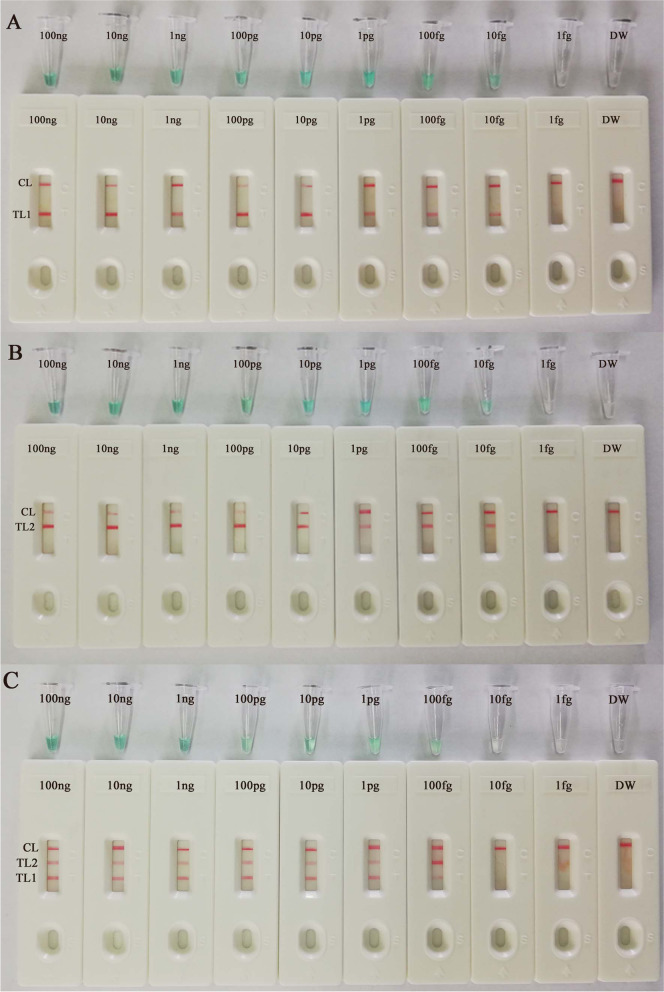
Sensitivity of the MCDA method using serially diluted genomic DNA extracted from *M. tuberculosis* (H37Rv, ATCC27294). A total of two detection techniques, including the lateral flow biosensor (LFB) and colorimetric indicator (MG) methods, were applied to analyze the amplification products. Serial dilutions of target templates were subjected to standard MCDA reactions. DNA levels of 100 ng, 10 ng, 1 ng, 100 pg, 10 pg, 1 pg, 100 fg, 10 fg and 1 fg per reaction. Double distilled water was the template for the blank control. **A** Singly, the *IS6110* gene at genomic DNA levels of 100 ng per reaction, 10 ng per reaction, 1 ng per reaction, 100 pg per reaction, 10 pg per reaction, 1 pg per reaction, 100 fg per reaction and 10 fg per reaction produced positive reactions. **B** The *mpb64* gene at genomic DNA levels of 100 ng per reaction, 10 ng per reaction, 1 ng per reaction, 100 pg per reaction, 10 pg per reaction, 1 pg per reaction, 100 fg per reaction and 10 fg per reaction produced positive reactions. **C** Both the *IS6110* gene and *mpb64* gene at genomic DNA levels of 100 ng per reaction, 10 ng per reaction, 1 ng per reaction, 100 pg per reaction, 10 pg per reaction, 1 pg per reaction and 100 fg per reaction produced positive reactions

The genomic DNA templates of *M. tuberculosis* (H37Rv, ATCC 27294) were serially diluted (100 ng, 10 ng, 1 ng, 100 pg, 10 pg, 1 pg, 100 fg, 10 fg and 1 fg per microliter) for MTBC-MCDA sensitivity analysis. The limiting dilution of *M. tuberculosis* genomic DNA was evaluated by MCDA detection. The single-MCDA-LFB assay limit of detection (LoD) targeting the *IS6110* gene (Fig. 4A) or *mpb64* gene (Fig. 4B) was found to be 10 fg per reaction. Double target genes were found to be 100 fg per reaction (Fig. 4C). As expected, CL, TL1 and/or TL 2 were observed on the biosensor, displaying positive MCDA results for the *IS6110* gene and/or *mpb64* gene. Double distilled water (DW) was the template for the blank control. Moreover, the analytical sensitivity of MTBC-MCDA with the biosensor was consistent with colorimetric indicator analysis.

### Specificity of the MTBC-MCDA-LFB assay

Thirty-eight MTBC strains, 12 NTM strains and 10 other bacteria were used to determine the specificity of the MCDA-LFB assay. When the genomic DNA of the bacteria listed in Table 2 was used for MCDA-LFB detection, only the DNA of the MTBC strains tested positive. The genomic DNA of NTM and other bacterial strains tested negative by the MCDA-LFB assay.

**Table 2:** The details of the strains

Bacteria	Strain no. (source of strains)	No. of strains	MCDA-LFB result
**MTBC**			
*M. tuberculosis*	H37RvATCC27294	1	**P**
*M. tuberculosis*	Isolated strains (GZCDC)	34	**P**
*M. bovis*	ATCC19210	1	**P**
*M. africanum*	ATCC25420	1	**P**
*Bacillus Calmette-Guerin*	Vaccine strain (GZCDC-BCG)	1	**P**
**NTM**			
*M. aureus*	ATCC23366	1	**N**
*M. flavum*	ATCC43999	1	**N**
*M. avium*	ATCC25291	1	**N**
*M. marinum*	ATACC927	1	**N**
*M. abscess*	ATCC19977	1	**N**
*M. chelonae*	ATCC14472	1	**N**
*M. gordon*	ATCC14470	1	**N**
*M. phlei*	ATCC11758	1	**N**
*M. nonchromogenic*	ATCC19530	1	**N**
*M. xenopi*	ATCC19250	1	**N**
*M. aichiense*	ATCC27280	1	**N**
*M.microti*	ATCC19422	1	**N**
**Other bacteria species**			
*Enterococcus faecalis*	Isolate strains (GZCDC)	1	**N**
*Salmonella*	Isolate strains (GZCDC)	1	**N**
*Klebsiella pneumoniae*	Isolate strains (GZCDC)	1	**N**
*Pseudomonas aeruginosa*	Isolate strains (GZCDC)	1	**N**
*Staphylococcus aureus*	Isolate strains (GZCDC)	1	**N**
*Escherichia coli*	Isolate strains (GZCDC)	1	**N**
*Bacillus cereus*	Isolate strains (GZCDC)	1	**N**
*Listeria monocytogenes*	Isolate strains (GZCDC)	1	**N**
*Streptococcus pneumoniae*	Isolate strains (GZCDC)	1	**N**
*Campylobacter jejuni*	Isolate strains (GZCDC)	1	**N**

*Abbreviations*: *MTBC Mycobacterium tuberculosis* complex, *NTM nontuberculous mycobacterium*, *ATCC* American Type Culture Collection, *GZCDC* Guizhou Provincial Center for Disease Control and Prevention, *P* positive, *N* negative

### Application of the MTBC-MCDA-LFB assay for sputum samples

Fifty-one sputum samples (provided by pulmonary hospital of Guiyang) were detected by acid-fast staining (AFS), conventional culture method, seminested automatic real-time PCR (Xpert MTB/RIF) and MCDA-LFB. The AFS results consisted of 26 positive samples and 25 negative samples, and the positive detection rate was 50.98%. Additionally, the sputum samples were cultured, and the results showed that 35 samples were positive and 16 were negative after 8 weeks. The Xpert MTB/RIF results indicated that 39 were positive and 12 were negative, and the rate of positive detection was 76.47%. The MCDA-LFB results showed that 41 were positive and 10 were negative, and the rate of positive detection was 80.39% (Table 3).

**Table 3:** Sputum samples and MTBC strain detection

Methods	Sputum samples (***N*** = 51)	
	Positive	Negative
Acid-fast staining	26	25
Culture	35	16
Gene-Xpert	39	12
MCDA-LFB	41	10

*Abbreviations: MCDA-LFB* multiple cross displacement amplification with lateral flow biosensor

## Discussion

To date, TB remains a chronic infectious disease caused by MTBC members that seriously endangers human health [1, 5]. Thus, convenient, rapid, sensitive and specific detection of MTBC is important for the prevention and treatment of TB, especially the development of novel rapid detection techniques. However, conventional detection methods (i.e., SSM, mycobacterial cultivation identification and biochemical tests) usually cannot meet the needs of rapid detection in terms of the detection period and sensitivity [6, 8]. PCR and PCR-based assay techniques displayed certain reliability and sensitivity in previous publications, but the requirements for PCR thermal cyclers and expensive reagents hindered their application in resource-poor areas [8]. Among the various rapid detection methods, MCDA, as a low-cost (the cost of a single MCDA reaction was estimated to be approximately 5 USD, and the LFB was 2.5 USD per test), highly sensitive and specific assay based on the LAMP technique [21], was established and applied for the detection of various pathogens (e.g., bacteria, viruses, and fungi) [21–23]. Currently, verification methods for MCDA amplicons have always been a major concern, especially for multiplex MCDA assays. Unfortunately, conventional methods (containing visual reagents, agarose gel electrophoresis and real-time turbidimetry) have difficulty verifying multiplex MCDA amplicons. Thus, LFBs based on nanoparticles were designed and utilized in our experiments. In this report, the MCDA technique combined with a nanoparticle-based LFB (MCDA-LFB) for rapid detection of MTBC was developed successfully.

In the current report, the *IS6110* and *mpb64* genes were chosen as specific molecular targets, and two sets of MCDA primers were designed. The *IS6110* sequence, which belongs to a family of ISs of the IS3 category with high specificity, has been widely applied for MTBC-PCR assays [24]. However, some *M. tuberculosis* strains lacking the *IS6110* gene were found in several clinical investigations [25, 26]. Thus, the *mpb64* gene, which encodes the RD2 region of the MPB64 protein in the genome of *M. tuberculosis* [27], was introduced in our study, and it showed excellent specificity for MTBC strains in previous studies [9, 27]. There is some evidence for the absence of the *mpb64* gene in some substrains of *Mycobacterium bovis* BCG [28]. However, there are no reports of the absence of both the *IS6110* and *mpb64* genes. In this study, we chose both the *IS6110* gene and *mpb64* gene as the target genes for MTBC to ensure its specificity. The MCDA-LFB technique targeting the *IS6110* and *mpb64* genes was successfully established and applied to the detection of MTBC. As Table 2 shows, 38 MTBC strains, 12 NTM strains and 10 other bacterial strains were used to test the specificity of the MCDA-LFB assay. The specificity of the MCDA-LFB assay was 100%, and there was no cross-reactivity with other pathogens (including NTM and nonmycobacterial strains).

In this research, the MCDA products were analyzed using the LFB, MG indicator and real-time turbidity methods. Comparing the above three validation methods, the LFBs were more convenient, reliable and visualized. They did not require special instruments or reagents [16, 17]. In addition, LFB can detect both the *IS6110* and *mpb64* genes and allow them to be visualized in a single test at the same time. According to the results, at 67 °C, both *IS6110* and *mpb64* gene amplification had higher turbidity and shorter reaction times at 30 min and 23 min (Fig. 2 and Table 1). The LFB assay indicated that the earliest test lines were observed for both TL1 and TL2 when the amplification lasted for 30 min at 67 °C, but the TL2 line was light red (Fig. 3). Hence, to ensure adequate amplification, the conditions of amplification temperature and time were optimized for MCDA-LFB at 67 °C for 35 min in this study. In addition, based on the *IS6110* and *mpb64* genes, the MCDA assay LoD was 100 fg of genomic DNA per reaction (Fig. 4). We found that the sensitivity of the single MCDA assay (10 fg per reaction) was higher than that of the multiplex MCDA assay (100 fg per reaction) in the sensitivity experiment. This phenomenon may be caused by the different concentrations of the single set of primers in the singlex and/or multiplex MCDA reaction system (e.g., the CP1 of *IS6110* primers was 2.4 μM in the singlex MCDA system and 1.2 μM in the multiplex MCDA system) [29].

For sputum sample detection, Xpert MTB/RIF and MCDA-LFB had higher positive rates than the conventional culture and acid-fast staining assays (Table 3). While this is a possible reason for culture method that there were no living MTBC strains in the sputum samples, the Xpert MTB/RIF and/or MCDA-LFB could detect the nucleic acid of the target strains. In addition, the positive rates of the Xpert MTB/RIF (76.47%, 39/51) and MCDA-LFB assays (80.39%, 41/51) for sputum specimens were similar (Table 3). Xpert MTB/RIF is an important tool for MTBC with rifampin (RIF) resistance [30], and the detection time is approximately 2.5 h. Usually, Xpert MTB/RIF has a four-channel detection system, which means that this machine can test 4 samples at a time and is not suitable for the large-scale detection of MTB samples. Moreover, Xpert MTB/RIF needs to use the matching kit, which represents a high cost. However, the MCDA-LFB technique was a better choice for detecting MTBC with a large specimen size. Importantly, it is low cost (approximately 7.5 USD per test). Compared with the AFS method, combined microscopy detected MTB of sputum specimens, an approach that lacks sensitivity [31, 32] and takes approximately 2 h, including fixing, dyeing, drying naturally and microscopy. Microscopy took more time as the samples increased. However, the MCDA-LFB technique can solve this problem well. The whole detection process was completed within 70 min, including genomic template preparation (approximately 30 min), MCDA reaction (approximately 35 min) and LFB verification (approximately 1–2 min), and the maximum detection quantity was 96 samples with a conventional PCR apparatus.

## Conclusions

In this study, a reliable, rapid, visual, inexpensive and simple MCDA-LFB assay based on the *IS6110* and *mpb64* genes was successfully established for the detection of MTBC. This technique could identify the *M. tuberculosis* complex with high specificity, sensitivity, and rapidity, and it was a visual and simple assay of the MCDA products. Thus, the MTBC-MCDA-LFB method could be regarded as a useful technique for rapid and reliable identification of the *M. tuberculosis* complex in clinical samples. Moreover, the technique did not require complicated instruments and expensive reagents and could be used widely, especially in resource-limited areas of developing countries with a high TB burden.

## Methods

### Reagents and apparatus

DNA isothermal amplification kits, colorimetric indicators (malachite green, MG), and biotin-14-dCTP were purchased from Bei-Jing HaiTaiZhengYuan. Co., Ltd. (Beijing, China). DNA extraction kits (QIAamp DNA minikits; Qiagen, Hilden, Germany) were purchased from Qiagen (Beijing, China). The nanoparticle-based lateral flow biosensor (Disposable Lateral Flow Biosensor, LFB) was manufactured by Tian-Jin HuiDeXin Biotech. Co., Ltd. (Tianjin, China).

### Design of MCDA primers

**Fig. 5:**
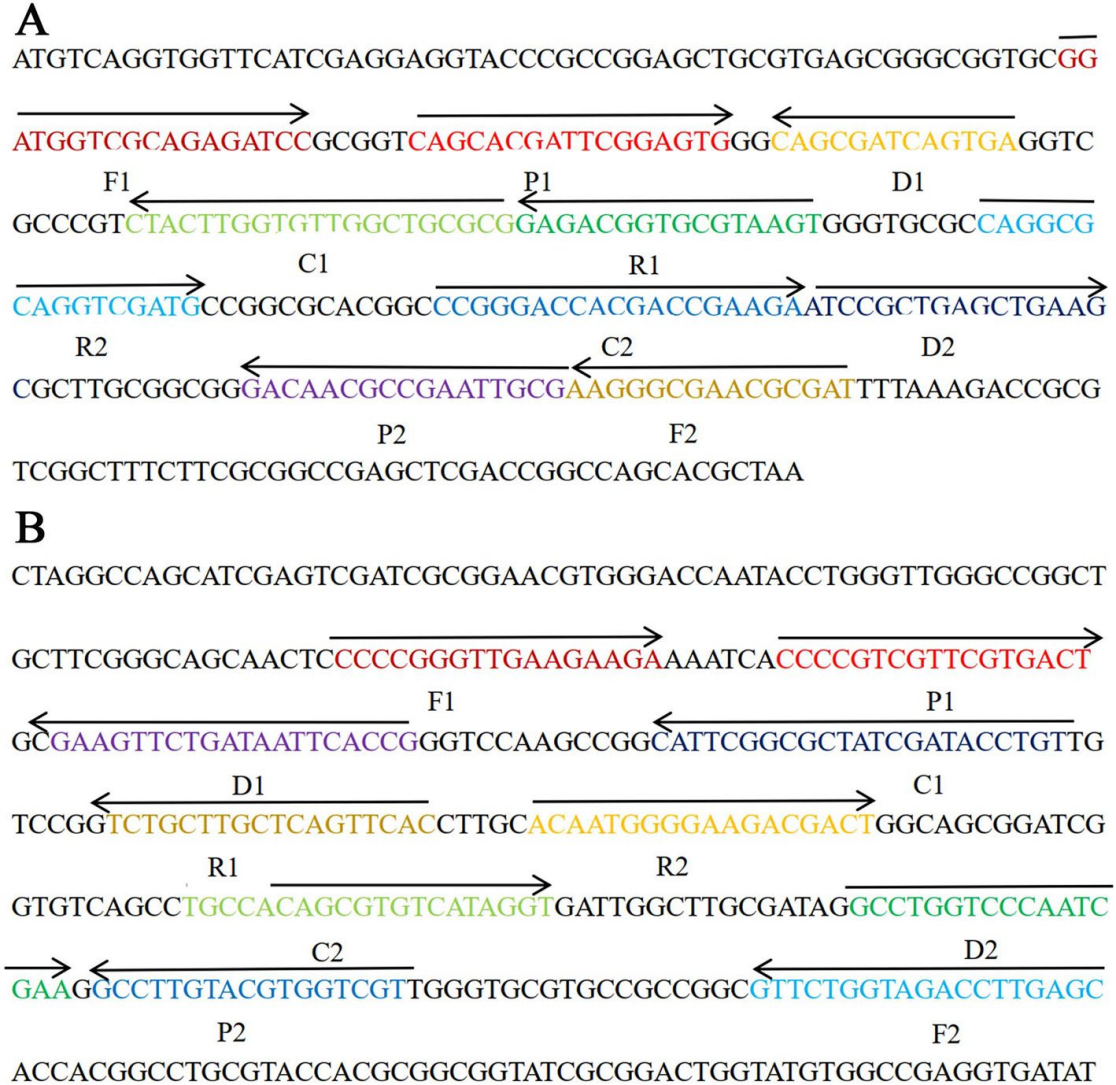
Sequences, modifications and locations in the expression sites of the *IS6110* and *mpb64* genes. **A** Location and nucleotide sequence of the *M. tuberculosis IS6110* gene used to design the MCDA primers. **B** Location and nucleotide sequence of the *M. tuberculosis mpb64* gene used to design the MCDA primers. All sequences of the primer sites are underlined. Right and left arrows indicate sense and complementary sequences that are used

Two specific MTC-MCDA target genes, *IS6110* (GenBank, Sequence ID: CP053903.1) and *mpb64* (GenBank, Sequence ID: CP053903.1), were chosen. The 10 specific primers were designed by primer software PRIMER PREMIER 5.0 and Primer Explorer V4 [15]. In addition, FITC (fluorescein isothiocyanate) was labeled at the 5′ end of the *IS6110*-C1* primer, and digoxigenin (Dig) was labeled at the 5′ end of the *mpb64*-C1* primer. The details, including primer design, sequences and modifications, and locations in the expression sites of the *IS6110* and *mpb64* genes are listed in Table 4 and Fig. 5. All of the primer sequences were synthesized and purified through Tian-Yi Biotech (Beijing, China) at HPLC purification grade.

**Table 4:** The details of primers for the *IS6110* and *mpb64* genes

Genes	Primers name ^**a**^	Sequences and modifications	Length
***IS6110***	IS6110-F1	5′-GGATGGTCGCAGAGATCC-3′	18 nt
	IS6110-F2	5′-ATCGCGTTCGCCCTT-3′	15 nt
	IS6110-CP1	5′-CGCGCAGCCAACACCAAGTAGCAGCACGATTCGGAGTG-3′	38 mer
	IS6110-CP2	5′-CCGGGACCACGACCGAAGACGCAATTCGGCGTTGTC-3’	36 mer
	IS6110-C1*	5′-FITC-CGCGCAGCCAACACCAAGTAG-3’	21 nt
	IS6110-C2	5′-CCGGGACCACGACCGAAGA-3’	19 nt
	IS6110-D1	5′-ACCTCACTGATCGCTG-3’	16 nt
	IS6110-D2	5′-ATCCGCTGAGCTGAAGC-3’	17 nt
	IS6110-R1	5′-ACTTACGCACCGTCTC-3’	16 nt
	IS6110-R2	5′-CAGGCGCAGGTCGATG-3’	16 nt
***mpb64***	mpb64-F1	5′-CCCCGGGTTGAAGAAGA-3’	17 nt
	mpb64-F2	5′-GCTCAAGGTCTACCAGAAC-3’	19 nt
	mpb64-CP1	5′-ACAGGTATCGATAGCGCCGAATGCCCCGTCGTTCGTGACT-3’	40 mer
	mpb64-CP2	5′-TGCCACAGCGTGTCATAGGTACGACCACGTACAAGGC-3’	37 mer
	mpb64-C1*	5′-Dig-ACAGGTATCGATAGCGCCGAATG-3’	23 nt
	mpb64-C2	5′-TGCCACAGCGTGTCATAGGT-3’	20 nt
	mpb64-D1	5′-CGGTGAATTATCAGAACTTC-3’	20 nt
	mpb64-D2	5′-GCCTGGTCCCAATCGAA-3’	17 nt
	mpb64-R1	5′-GTGAACTGAGCAAGCAGA-3’	18 nt
	mpb64-R2	5′-ACAATGGGGAAGACGACT-3’	18 nt

*Abbreviations: FITC* fluorescein isothiocyanate, *Dig* digoxigenin, *mer* monomeric unit, *nt* nucleotide

^a^ IS6110-C1*, 5′-labeled with FITC when used in the MCDA-LFB assay; mpb64-C1*, 5′-labeled with Dig when used in the MCDA-LFB assay

### Bacterial strains and genomic DNA preparation

There were 60 strains, including 38 MTBC strains, 12 nontuberculous mycobacterium (NTM) strains and 10 other bacteria, and the details are shown in Table 2. The bacterial strains were stored in 10% (w/v) glycerol broth at − 80 °C and then revived and cultured. According to the instruction book, the genomic templates were extracted from the cultured strains by the QIAamp DNA Mini Kit (Qiagen, Germantown, MD, USA). Sputum samples were treated with 4% NaOH solution, and genomic templates were extracted by the kit. Then, the genomic templates were tested by ultraviolet spectrophotometry at A260/280 and stored at − 20 °C before the templates were used.

### MCDA reaction and detection

The suitability of two target genes for the MCDA primers was examined through MCDA reaction of the single *IS6110* gene for the *mpb64* gene. Then, both target genes were detected at the same time. Genomic templates of the standard strain *M. tuberculosis* (H37Rv, ATCC 27294) were used for the MCDA assay according to the DNA isothermal amplification kits. Briefly, the MCDA reaction mixtures contained 12.5 μl of 2 × Buffer, 1 μl of Bst 2.0 DNA polymerase (10 U), 1 μl of biotin-14-dCTP, 1 μl of malachite green, 2 μl of genomic templates, 5.3 μl of DW and 2.2 μl of amplification primers or mixed primers containing 0.4 μM each of displacement primers F1 and F2, 0.4 μM each of amplification primers C1* and C2, 1.2 μM each of amplification primers R1, R2, D1 and D2, and 2.4 μM each of cross primers CP1 and CP2 in a final volume of 25 μl. Then, they were reacted for 35 min at 67 °C. Three detection methods, including a real-time turbidimeter (LA-320C), disposable lateral flow biosensor (LFB) and colorimetric indicator (MG), were applied to detect the MCDA amplification products.

The threshold value (turbidity) was 0.1, and a turbidity of > 0.1 was regarded as positive amplification for the MCDA-LFB assay by using a real-time turbidimeter [16]. The LFB consisted of the sample pad, conjugate pad, NC membrane, and absorbent pad, which were laminated onto a plastic adhesive backing card. Then, the anti-FITC Ab, anti-Dig Ab and biotin-BSA were sprayed onto the NC membrane for TL1, TL2 and CL, respectively [19]. The detection method of LFB involves depositing an aliquot (1–2 μl) of MCDA amplification products on the sample pad of LFB and then depositing an aliquot of running buffer (150–200 μl) on the sample pad of LFB. The amplicons (crimson red) were captured by specific antibodies (namely, anti-FITC Ab, anti-Dig Ab, and biotin-BSA) when flowing through the NC membrane. The red lines (i.e., CL, TL1 and/or TL2) were observed in the positive amplification, while only CL was red in the blank and negative controls. In addition, the color of positive amplification changed from blue to light blue, while the blank and negative controls were colorless by using MG indicators. In this study, DW was used as the template for the blank control, and the genomic DNA of *M. avium* was used as the template in the negative control.

### Optimizing the reaction temperature and time of the MCDA assay

Both the *IS6110* gene and *mpb64* gene were detected at different temperatures by a real-time turbidimeter (LA-320C). We examined the effect of different temperatures, from 63 to 70 °C, with 1 °C intervals for MCDA amplification. The *M. avium* genomic templates were used as a negative control. Subsequently, the two target genes were confirmed at different amplification reaction times, 20, 30, 40 and 50 min, detected by LFBs and MG.

### Analytical sensitivity and specificity of MTBC-MCDA-LFB assay

The genomic DNA templates of MTB (H37Rv, ATCC 27294) were serially diluted (100 ng-1 fg per microliter) for sensitivity analysis by MCDA-LFB detection for single genes and double genes. DW was the template for the blank control. Thirty-eight MTBC strains, 12 NTM strains and 10 other bacteria (Table 2) were used for the specificity assay. The genomic DNA from the strains was amplified by MCDA reactions and then assayed through LFB. The experiments were repeated at least two times.

### Practicability of the MTBC-MCDA-LFB assay for sputum samples

Fifty-one sputum samples provided by the Pulmonary Hospital of Guiyang (China) were detected by AFS, conventional culture, Xpert MTB/RIF and MTBC-MCDA-LFB methods. The AFS method was performed in our study according to previous publications [32]. Meanwhile, Xpert MTB/RIF and conventional culture methods were implemented according to the manufacturer’s instructions. The MTBC-MCDA assay was carried out as described above, and its amplicons were validated by LFBs.

## Data Availability

The datasets used and/or analyzed during the current study are available from the corresponding author upon reasonable request. The raw sequence data reported in this paper came from GenBank, Sequence ID: CP053903.1, *M. tuberculosis* strain H37Rv_IC1chromosome (https://www.ncbi.nlm.nih.gov/nuccore/CP053903.1?report=fasta).

## Abbreviations

MTBC: *Mycobacterium tuberculosis* complex
TB: Tuberculosis
MCDA: Multiple cross displacement amplification
LFB: Nanoparticle-based lateral flow biosensor
AFS: Acid-fast staining
WHO: World Health Organization
FITC: Fluorescein isothiocyanate
Dig: Digoxigenin. mer, monomeric unit
nt: Nucleotide
ATCC: American Type Culture Collection
LoD: Limit of detection
MG: Malachite green
GZCDC: Guizhou Provincial Center for Disease Control and Prevention
NTM: *Nontuberculous mycobacterium*
P: Positive
N: Negative
TL1: Test Line 1
TL2: Test Line 2
CL: Control line
DW: Double-distilled water
BC: Blank control
PC: Positive control
NC: Negative control
